# Locomotion Guidance by Extracellular Matrix Is Adaptive and Can be Restored by a Transient Change in Ca^2+^ Level

**DOI:** 10.1371/journal.pone.0007330

**Published:** 2009-10-05

**Authors:** Hong-Wen Liu, Yun-Cin Luo, Chia-Lin Ho, Jung-Yen Yang, Chi-Hung Lin

**Affiliations:** 1 Institute of Microbiology and Immunology, National Yang-Ming University, Taipei, Taiwan; 2 Institute of Biophotonics, National Yang-Ming University, Taipei, Taiwan; 3 National Nano Device Laboratories, Hsinchu, Taiwan; 4 Department of Surgery, Veteran General Hospital, Taipei, Taiwan; 5 Taipei City Hospital, Taipei, Taiwan; University of Birmingham, United Kingdom

## Abstract

Navigation of cell locomotion by gradients of soluble factors can be desensitized if the concentration of the chemo-attractant stays unchanged. It remains obscure if the guidance by immobilized extracellular matrix (ECM) as the substrate is also adaptive and if so, how can the desensitized ECM guidance be resensitized. When first interacting with a substrate containing micron-scale fibronectin (FBN) trails, highly motile fish keratocytes selectively adhere and migrate along the FBN paths. However, such guided motion become adaptive after about 10 min and the cells start to migrate out of the ECM trails. We found that a burst increase of intracellular calcium created by an uncaging technique immediately halts the undirected migration by disrupting the ECM-cytoskeleton coupling, as evidenced by the appearance of retrograde F-actin flow. When the motility later resumes, the activated integrin receptors render the cell selectively binding to the FBN path and reinitiates signaling events, including tyrosine phosphorylation of paxillin, that couple retrograde F-actin flow to the substrate. Thus, the calcium-resensitized cell can undergo a period of ECM-navigated movement, which later becomes desensitized. Our results also suggest that endogenous calcium transients as occur during spontaneous calcium oscillations may exert a cycling resensitization-desensitization control over cell's sensing of substrate guiding cues.

## Introduction

Cell locomotion may be navigated by gradients of soluble factors [1]. Similar to chemotaxis by microorganisms [2], chemotactic migrations by neuronal cells [3] or neutrophils [4] can be desensitized in the constant presence of a chemo-attractant. To restart the inactivated guidance, the cell needs to periodically sample the concentration of chemo-attractant and integrate this with the signaling processes so that it resets the cell movement according to the environmental change [5]. In addition to soluble chemo-attractants, the chemistry and topography of a growth substrate to which the cells have attached can also control cell motility [6]. For example, at the boundary of different extracellular matrix (ECM) coatings, cells tend to differentiate and selectively adhere to the area where the preferred ECM molecules reside [7]. On a substrate fabricated with a gradient of growth-promoting ECM, neuronal growth cones can navigate up the concentration gradient [8]. However, in all these experimental settings, there has been no clear answer as to whether or not ECM-guided motility can become adaptive and if so, how can the desensitized ECM-guidance be reactivated. Here, we grow fish keratocytes onto substrates that are coated with micro-scaled paths of fibronectin (FBN), which enables us to assess ECM navigation by analyzing different motile behaviors as the cells crawling along or across the FBN paths. We found that when first encountering with an ECM-patterned substrate, fish keratocytes can selectively adhere and migrate along the FBN paths only for a limited period of time, then the cell's response to substrate guidance becomes adaptive or desensitized. Thirty min after plating, most fish keratocytes have moved out of the FBN path confinement and are undergoing undirected random migration. Interestingly, a calcium transient created by the calcium uncaging technique, or indeed from the native intracellular calcium oscillations, can re-sensitize an adaptive cell and render it responsive, for another short period of time, to substrate ECM guidance. Mechanisms underlying the adaptation and the calcium-induced resensitization-desensitization process are explored.

## Results and Discussion

### After a transient ECM-directed movement, the fish keratocyte adapts and becomes desensitized to substrate guiding cues and undergoes random migration

**Figure 1:**
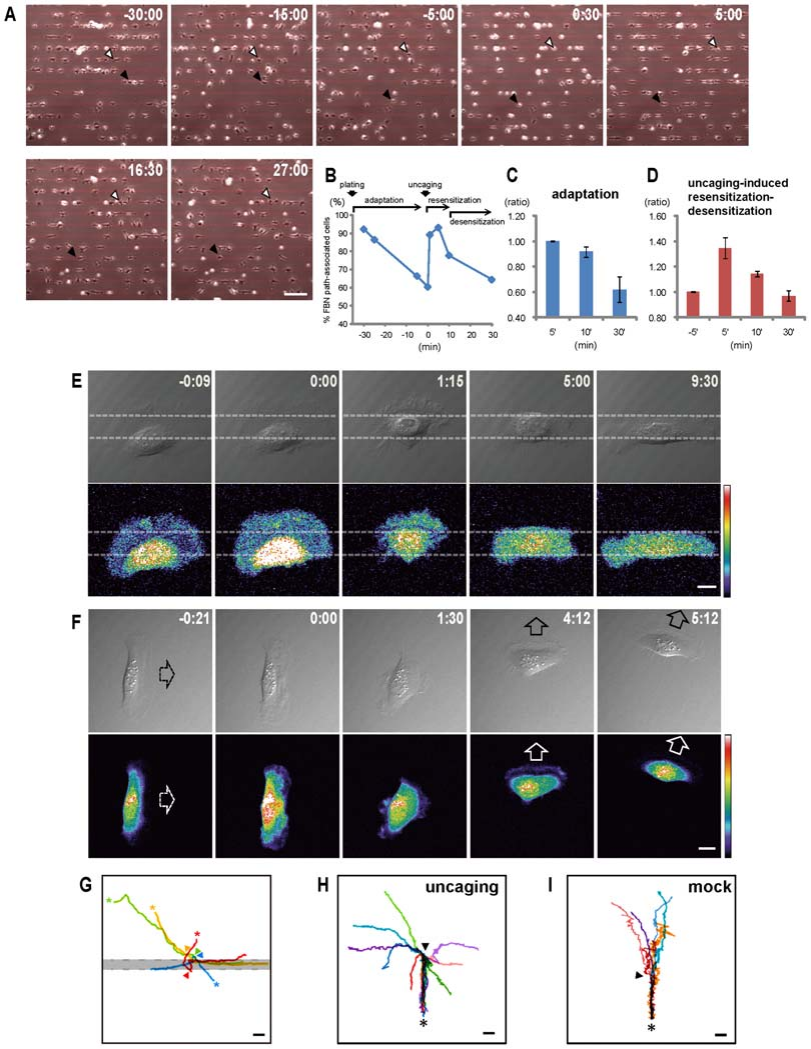
A calcium transient created by uncaging converts undirected cell migration into a brief period of ECM-guided movement. (A) A representative image sequence for adaptation to guided-motion and calcium-induced resensitization-desensitization process recorded under phase contrast microscopy; see also Movie S1. Right after being plated onto the substrate containing FBN paths (*red lines*), most fish keratocytes initially bind and move along the FBN paths (-30:00), but later leave the FBN path confinement (-15:00 and -5:00). After calcium uncaging done at 0:00, many cells are resensitized and become responsive to the substrate guiding cues again. These cells exhibit a period of re-association with FBN paths (0:30 and 5:00) before moving out of the paths (16:30 and 27:00). *Arrows* mark the same cells. Time (min:sec) before and after calcium uncaging is labeled as negative and positive values, respectively. (B–D) FBN path-associated cells, in which >50% of the cell area locates over the FBN path, are counted for the image sequence of Movie S1 (B), and for the averaged values obtained from three independent experiments, each counting >400 cells (C–D). FBN path-associated cells are shown in percentage (B), or as ratios normalized by the number taken at 5 min after plating (C) or 5 min before uncaing (D). (E) DIC and pseudo-colored calcium imaging sequence of a cell already adaptive to substrate guidance to become resensitized and re-associate with the FBN path (bounded by *dashed lines*) by the calcium burst generated at 0:00 by uncaging. (F) DIC and calcium imaging sequence of a cell changing its direction of movement (from *dashed arrow* to *open arrow*) on a homogenous FBN substrate following calcium uncaging at 0:00. See also Movie S2. (G) On the substrate containing FBN paths (*gray bar*), the cells about to traverse the ECM trail (*asterisks*) are subjected to calcium uncaging (done at *triangles*), that changes the cells' movements from crossing to crawling along the FBN path. (H–I) On a homogenous FBN substrate, migration tracks for individual cells recorded before (from *asterisks* to *triangle*) and after (tracks after *triangle*) uncaging are analyzed. Changes of movement directions are evident in cells receiving calcium uncaging (H), but not when mock photolyses are applied (I). Bar = 100 µm (A) or 10 µm (E–I).

One major difficulty facing the assessment of ECM-guidance is the lack of a convenient way to determine if the cell motion observed is guided by the substrate or not; this is because most substrates that are used are coated homogenously with ECM. To tackle this problem, we have employed a micro-contact printing technique in this study to deposit FBN as 10 µm-wide linear trails on a cover-glass substrate (*red bars*, Fig. 1A and area bounded by *dashed lines*, Fig. 1E). In the primary culture, these spatial features of ECM coating are able to last for more than 24 hr. As exemplified in Fig. 1A and Movie S1, fish keratocytes when first encountering such ECM-patterned substrates were able to recognize and respond to substrate guiding cues by preferentially adhering, spreading and migrating along the FBN paths (-30:00, see also [9]). However, such guided morphogenesis and motion was transient. Starting at around 10 min after plating, the cells began to adapt to substrate guidance, when they often ignored and moved outside of ECM coating confinement and sometimes traversed FBN path (-15:00 and -5:00). We quantified the cell's response to substrate guidance by counting FBN path-associated keratocytes, which were the cells in which >50% of their cell areas located on top of the patterned ECM coating. The changes of % FBN path-associated cells among all cells in the same image field during the process of adaptation are shown for the dynamic sequence of Movie S1 (Fig. 1B), and for the averaged values obtained from three independent experiments (Fig. 1C). In both cases, progressive decreases of cells' associations with the FBN paths after initial cell plating were evident.

### A burst of calcium increase in the fish keratocyte transiently resensitizes the cell to become responsive to substrate guidance, converting random migration to guided movement

Many experimental approaches were tried with the aim of converting fish keratocyte's adaptive random migration into ECM path-guided movement. We found that a burst increase in intracellular calcium created by calcium uncaging (or photolysis of a caged-calcium compound), when applied to a single fish keratocyte using a focused laser beam or to a selected population of cells using wide-field illumination, could successfully change the undirected motion into ECM-guided movement. As shown in Fig. 1A, uncaging done at 0:00 to the keratocytes that were already adaptive to the patterned ECM substrate (i.e., >30 min after plating) enabled many of them to resensitize and response to substrate guidance again by preferentially spreading and migrating along the FBN path (0:30 and 5:00). Such calcium-induced resensitization to substrate guiding cues was transient; the resensitized cells later became desensitized and moved out of the FBN path and underwent undirected migration (16:30 and 27:00). During such a calcium-induced resensitization-desensitization process, the trend of a transient increase followed by a decrease in the number of FBN path-associated cells was obvious for the sequence of Movie S1 (Fig. 1B), and for averaged values obtained from three independent experiments (Fig. 1D).

Under high magnification and calcium imaging microscopy (Fig. 1E), we noticed an immediate halt of undirected cell movement right after the single controlled release of intracellular “calcium burst”. When the standing cell gradually became motile again, the resumption of cell motion was in a sequence very similar to the guided movement undertaken by the cells when they first interacted with the linear FBN path. The change of cell motility was not due to the UV illumination used by uncaging as mock photolysis did not affect the random migration of the cells (data not shown). In Fig. 1G, cell migration tracks before (from *asterisks* to *triangle*) and after (after *triangle*) calcium uncaging are analyzed among cells that were approaching the FBN path from different directions. We found that no matter from which angle the cell was about to traverse the FBN trail, the burst increase of calcium was able to convert the crossing event into motion guided by the FBN path. This suggests that the calcium increase was able to transiently resensitize and render the cell responsive to the substrate navigation and reinitiated a period of ECM-directed movement.

The calcium burst could also “***reset***” the direction of cell migration on substrates consisting of homogenous FBN (Fig. 1F and IH). Following calcium uncaging, the cell stopped advancing; when the motility resumed, it often took a new direction (*open arrow*, Fig. 1F; see Movie S2) that differed from the original one (*dashed arrow*). As a control, mock photolysis did not influence either the rate or direction of ongoing cell movement (Movie S3). Pooled migration track analyses revealed the presence of clear re-orientation of cell movement caused by calcium uncaging (Fig. 1H), but not by mock photolysis (Fig. 1I).

### A burst increase of calcium activates integrin receptors and modulates cell's adherence to the ECM substrate

**Figure 2:**
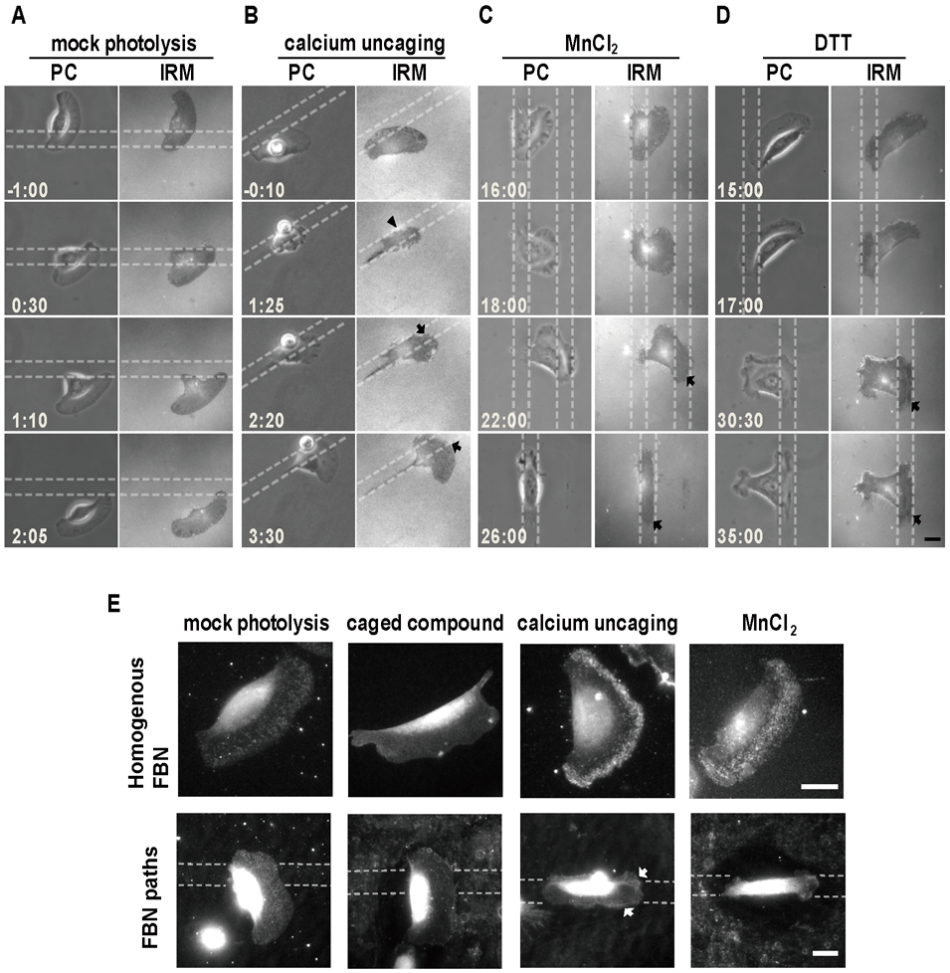
The created calcium transient reinitiates ECM-guided movement by reactivating integrins' recognition of the patterned ECM coating. (A–D) Imaging sequences (min:sec) under phase contrast (PC) or IRM microscopy. The cells already adaptive to substrate guidance are exposed to various treatments as indicated at 0:00. The cell receiving mock photolysis continues to move across the FBN path, exhibiting typical IRM patterns of a randomly migrating cell (A). The cell receiving calcium uncaging (B) first detaches from the substrate, evidenced by the appearance of IRM-bright zone (*arrowheads*), then re-adheres to the substrate, evidenced by the IRM dark area (*arrow*), as the cell advanced along the FBN trail. See also Movie S4. The cells treated with 5 mM MnCl_2_ (C), or 10 mM DTT (D) exhibited progressively increased cell adhesion (or IRM darkness); however, such chemical treatments, unlike calcium uncaging, have a long term inhibitory effect to cell movement. See also Movie S5 and S6. (E) Subcellular distributions of active-form integrin_β1_ are visualized by immuno-fluorescence staining in cells grown on the substrates containing homogenous FBN or FBN trails. Five min after calcium uncaging or 30 min after MnCl_2_ treatment, activated integrin_β1_ is noted to increase and/or preferentially accumulates at the cell's leading lamella (*arrow*), while mock photolysis or caged compound treatment alone has no obvious effect. Bar = 10 µm.

Interference reflection microscopy (IRM) was used to examine the live cells' interactions with substrates. When the motile fish keratocytes were examined, the IRM dark zones were found to associate with the leading edge of the lamella or where the adhesion complexes were located [10]. In the mock photolysis experiment (Fig. 2A), the IRM imaging sequence of a cell crawling across a FBN path did not differ from that of a control cell undergoing random migration. In the calcium uncaging experiment (Fig. 2B), the calcium transient caused the cell to “***detach***” from the substrate as evidenced by the appearance of an IRM-bright zone (*arrowhead*); this event coincided with the cessation of cell movement. When the stationary keratocytes later moved along the FBN path, the IRM-dark zones reappeared at the leading lamella of the cells (*arrows*, Fig. 2B and see Movie S4).

The reappearance of IRM darkness when motility resumed suggested that the cells were becoming more “adhesive” to the ECM substrate. This could happen if the calcium transient somehow activated the integrin receptors. To test this possibility, we performed immuno-fluorescence staining to localize active-form integrin_β1_ before and after calcium uncaging. As shown in Fig. 2E, the cells receiving either mock photolysis or caged compound loading alone contained only low abundance of active-form integrin_β1_ over the entire lamellar region. On the other hand, calcium uncaging for just 5 min was able to increase and/or redistribute active-form integrin_β1_; more staining signal was noted at the lamella near the leading edge or where the lamella interacted with the FBN path than elsewhere (*arrow*, Fig. 2E). Dithiothreitol (DTT) or MnCl_2_ have been shown to “activate” integrins by causing conformational changes of the integrin molecule [11]. Indeed, treating fish keratocytes with MnCl_2_ resulted in a profound increase in active-form integrin_β1_; however, the increased active-form integrin_β1_ induced by such a chemical was found to be located over the entire lamella (Fig. 2E), rather than near the leading edge, which was the situation when calcium uncaging was applied.

### A calcium burst reinitiates ECM-cytoskeleton coupling that modulates the conversion of retrograde F-actin flow into forward cell movement

**Figure 3:**
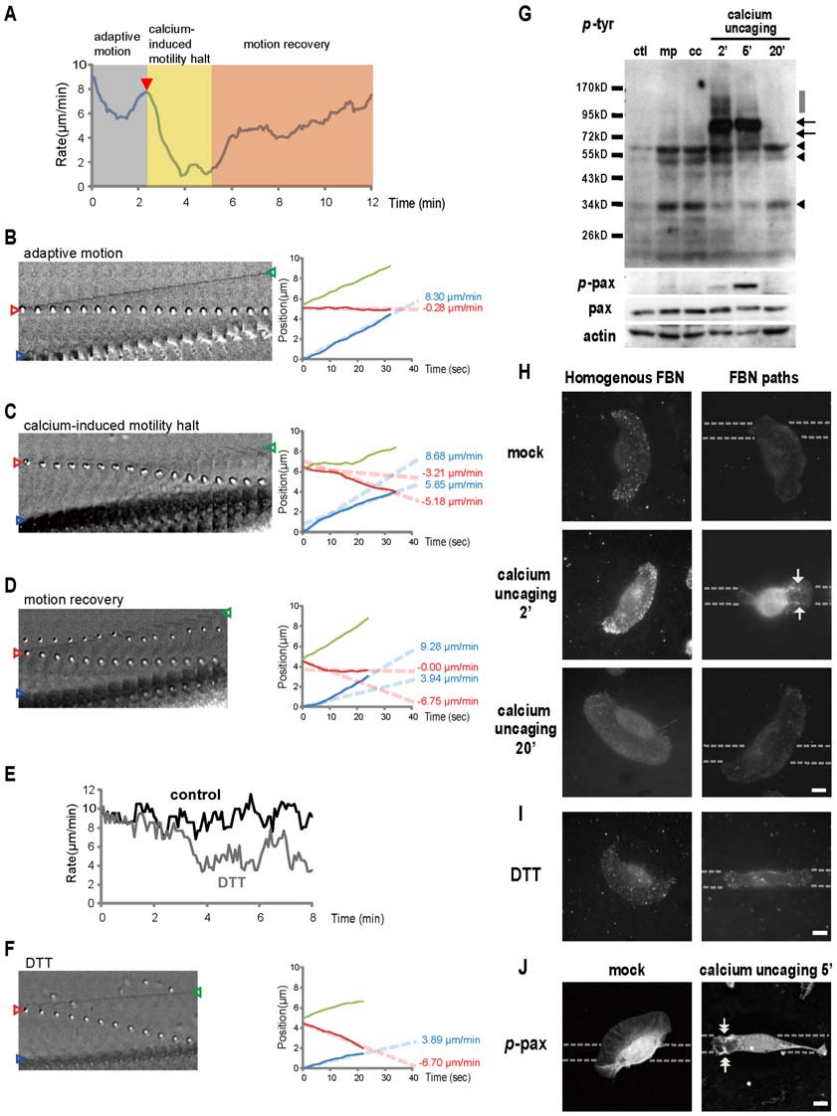
The calcium transient, through modulating retrograde F-actin flow's coupling to the substrate, leads to a period of ECM-guided motion. (A) Rates of cell movement are measured over time in a typical calcium uncaging experiment. Before uncaging, the cell is undergoing fast but undirected cell movement (*gray zone*). Photolysis is applied at *red triangle*. During the process of calcium-induced re-sensitization, the cell motility initially declined (*yellow zone*), then recovered (*orange zone*). (B–D) Positively charged 600-nm beads bound to the lamella reflect the dynamics of retrograde F-actin flow (*red traces and open triangle*). Rates of cell advance are measured by the extension of leading edge (*green traces and open triangle*), as well as organelle-rich cytoplasmic domain (*blue traces and open triangle*). Time interval for the image tile is 2 sec. (E) Rates of cell movement are monitored over time before (*black trace*) and after (*gray trace*) DTT treatment. (F) Retrograde F-actin flow is evident when the DTT treatment slows down cell advance. (G) Western blotting analyses for phosphorylated tyrosine (*p*-tyr), phosphorylated paxillin (*p*-pax), paxillin (pax) and actin are done in control cells (ctl), cells receiving mock photolysis (mp) or caged compound loading alone (cc), or cells incubated for 2 min, 5 min and 20 min after calcium uncaging. (H–I) Immunofluorescence staining of *p*-tyr in cells receiving the treatments as indicated. Staining of *p*-tyr increases/accumulates at the leading lamella of the cell crawling along the FBN path (*arrow*) at 2 min after calcium uncaging, then decreased at 20 min. (J) Immunofluorescence staining of *p*-pax in cells receiving mock photolysis or 5 min after calcium uncaging. Accumulation of *p*-pax was noted after uncaging at the leading lamella (*double arrows*). Bar = 10 µm (H–J).

**Figure 4:**
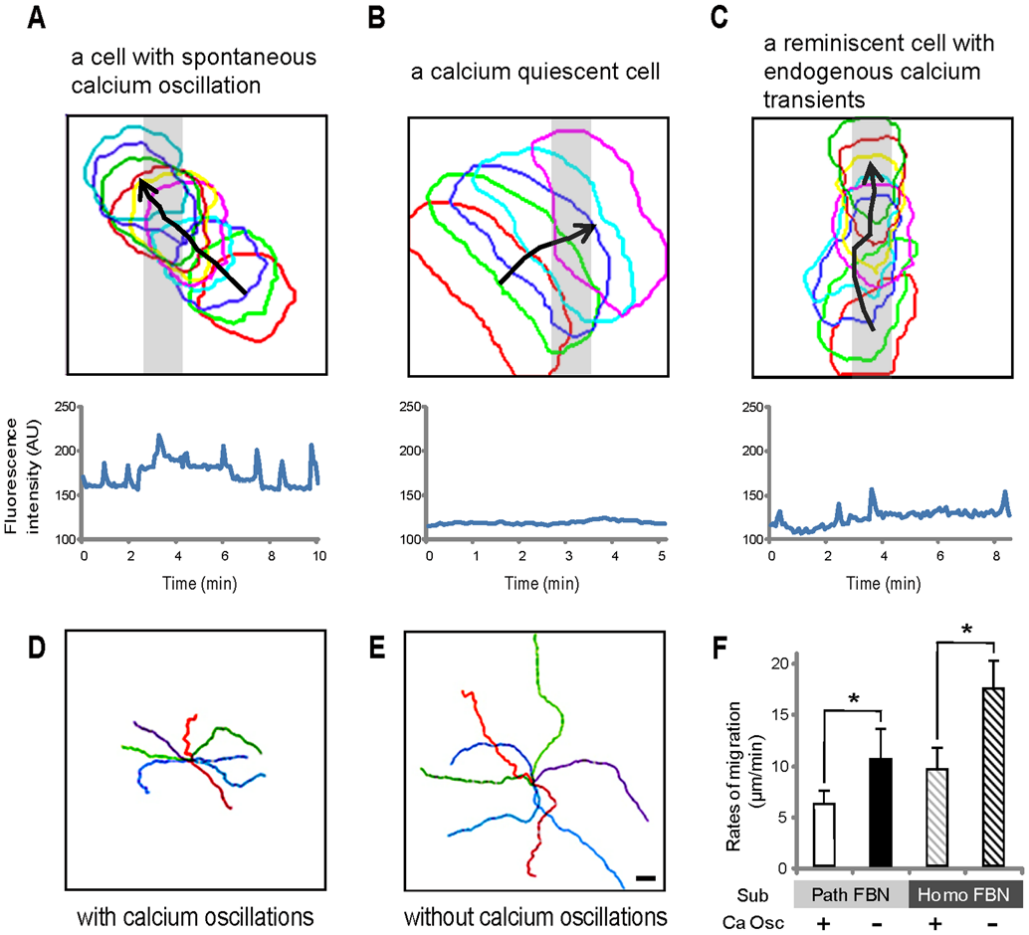
Motility dynamics differ between cells exhibiting spontaneous calcium oscillations and cells that are quiescent in calcium transient. (A–C) The sequences of cell contour tracing taken every 5 sec and the concurrent calcium imaging result recorded as a fish kearatocyte, already adaptive to substrate guidance, interacting with a FBN path (*gray bar*). *Black arrowed lines* link the geometric centers of individual cell contours. Note the keratocyte having calcium oscillation (A) traverses a FBN path at a much slower rate than the calcium-quiescent cell (B). A calcium-oscillating keratocyte can also undertake a brief period of guided motion and crawls along the FBN path (C); this never occurs in the calcium-quiescent cell observed. Bar = 10 µm. (D–E) Migration tracks for 10 min are displayed for the cells showing spontaneous calcium oscillation (D), or the cells treated with calcium chelators that eliminate all endogenous calcium transients (E). Bar = 20 µm. (F) Averaged rates of cell movement on either FBN paths or homogenous FBN (Homo FBN) are calculated among the cells having spontaneous calcium-oscillations, or being treated with calcium chelators. * indicates P<0.0001 by t-test. Substrate (Sub); calcium oscillations (Ca Osc).

Unlike the calcium transient that subsequently led to ECM-guided cell movement, MnCl_2_ or DTT treatment stops locomotion and the cell remained stably adhesive to the FBN path for a long period of time (*arrows*, Fig. 2C, D and see Movie S5, 6). Previous studies have shown that when cells are advancing, there is tight regulation by a coupling mechanism that converted retrograde F-actin flow into forward cell movement [12]. We therefore compared the F-actin flow dynamics between the cells treated with calcium uncaging, to the cells exposed to MnCl_2_ or DTT treatment. The centripetal transport of positively charged micro-particles adhered to the cell surface, by coupling to the intracellular actin network, is indicative of retrograde F-actin flow [13], [14]. Fig. 3A shows the dynamic of cell movement rates plotted as a function of time in a typical calcium uncaging experiment. At key time points (Fig. 3B–D), the rates of cell advance were measured by tracing both lamella leading edge (*green traces and open triangle*) and organelle-rich proximal cytoplasmic domain (*blue traces and open triangle*), and the rates of concurrent retrograde F-actin flow by the microbeads (*red traces and open triangle*). During the phase of adaptive motion (*gray zone* of Fig. 3A, and Fig. 3B), the cell moved forward at an averaged rate of 8.30 µm/min, but the retrograde F-actin flow rate was only 0.28 µm/min. Right after calcium uncaging (*red triangle*) in the phase of calcium-induced resensitization, forward cell movement initially reduced (*yellow zone* of Fig. 3A and Fig. 3C); when the rate of cell advance declined from 8.68 µm/min to 5.85 µm/min, the corresponding rate of retrograde F-actin flow increased from 3.21 µm/min to 5.18 µm/min (Fig. 3C). The slowed motility later recovered (*orange zone* of Fig. 3A and Fig. 3D); when the cell advance drastically increased from 3.94 µm/min to 9.28 µm/min, retrograde F-actin flow decreased from 6.75 µm/min to almost zero. Treating the cells with DTT also caused a progressive decrease in cell movement from 8∼10 µm/min before drug treatment (*black trace*, Fig. 3E) to 4∼6 µm/min after drug exposure (*gray trace*). Thirty min after addition of DTT, when the rate of cell advance reduced to 3.89 µm/min, retrograde F-actin flow appeared at a rate of 6.70 µm/min (Fig. 3F). However, the decreased motility caused by DTT never recovered and the robust retrograde F-actin flow continued. These results suggested that although DTT or MnCl_2_ could convert the integrins into an active-form conformation that bound to ECM, such chemically activated integrins were unable to re-establish ECM-cytoskeleton coupling, thereby locking retrograde F-actin flow at a persistent slippery state that failed to drive cell advance.

### The calcium-induced protein phospholylation is involved in controlling the ECM-cytoskeleton coupling

The reverse relationship between the rates of retrograde F-actin flow and forward cell movement (Fig. 3A–D) supports the notion that the calcium transient might regulate ECM-cytoskeleton coupling and thereby control cell motility. Several lines of evidence suggested that tyrosine phosphorylation, especially at certain actin-binding proteins, is involved in the modulation of cell motion [15]. We therefore carried out Western blotting analyses using antibodies made against phosphorylated tyrosine (*p*-tyr) on cell subjected to calcium uncaging. As shown in Fig. 3G, we found that neither the loading of the caged compound alone (***cc***), nor mock photolysis (***mp***) significantly affected the *p*-tyr blotting pattern. In contrast, calcium uncaging caused specific and characteristic changes in *p*-tyr protein profiles. We found that certain proteins for which the *p*-tyr dramatically increased in the first 5 min after the calcium transient, but then returned to the control level after 20 min (*arrows*). Furthermore, increases in *p*-tyr for some proteins lasted even shorter than 5 min (*gray bar*). There were also proteins, the *p*-tyr of which progressively decreased in response to the calcium transient (*arrowhead*).

Immunofluorescence staining (Fig. 3H) also showed that *p*-tyr signals were significantly increased or redistributed by the calcium transient. In cells grown on homogenous FBN substrates, the increased *p*-tyr was located over most of the extending lamella, while in cells grown on FBN paths, more *p*-tyr were found to have accumulated at the leading lamella that was crawling along the FBN path (*arrow*) than elsewhere. The accumulation of *p*-tyr was most evident at 2 min after calcium uncaging, but had diminished greatly after 20 min. Interestingly, DTT treatment did not cause any significant increase or accumulation of *p*-tyr staining as compared to the control cells (Fig. 3I).

In leukocytes, the actin-binding protein paxilin has been shown to connect integrin receptors to actin cytoskeleton through protein phosphorylation control [16]. Therefore, we next examined the phosphorylated paxillin (*p*-pax) content of the cells after calcium uncaging. As shown in Fig. 3G, we found that although the amount of paxillin protein did not significantly change, the protein's phosphorylation level strongly increased in the first 5 min after the calcium transient, but had diminished after 20 min. Immunofluorescence staining of *p*-pax (Fig. 3J) also revealed an apparent *increase/redistribution* of *p*-pax at 5 min after calcium uncaging. Accumulation of *p*-pax at the leading lamella that guided the cell movement along the FBN path (*double arrow*) was noted, as compared to the dim and homogenously distributed *p*-pax across the entire lamella in the control cell receiving mock photolysis.

### Spontaneous calcium oscillations periodically reinitiate substrate recognition and modulate motility accordingly

The next question to ask was whether the phenotype of calcium uncaging described here might be extended to the physiological control of cell motility by native calcium signaling. To explore this question, we carefully examined the pattern of cell motion and correlated the dynamics of cell movement with the occurrences of spontaneous calcium transients. Typical examples of this series of study are shown in Fig. 4. Although an adaptive fish keratocyte showing active calcium oscillations could still traverse the FBN path (Fig. 4A), the movement of crossing was slow and the cell appeared to be “dragged” along the way by the substrate, as compared to the fast crossing motion exerted by the adaptive cell showing no calcium transient (Fig. 4B). Some of the calcium oscillating cells did occasionally undergo short periods of directed movement along the FBN path as exemplified in Fig. 4C, in a sequence that was reminiscent to the guided motion following calcium-induced resensitization. On the homogenous FBN substrate, the migration tracks were compared between the control cells exhibiting calcium oscillations (Fig. 4D) and the cells whose calcium oscillations were inhibited by loading the cells with calcium chelators (Fig. 4E). We found that the calcium oscillating cells moved around and covered an area that was much smaller than the calcium quiescent cells. Similarly, rates of cell migration (on substrates coated with either FBN paths or homogenous FBN) were significantly higher in cells whose calcium transients were inhibited by loading the cells with calcium chelators, than the control cells that had calcium oscillations (Fig. 4F).

**Figure 5:**
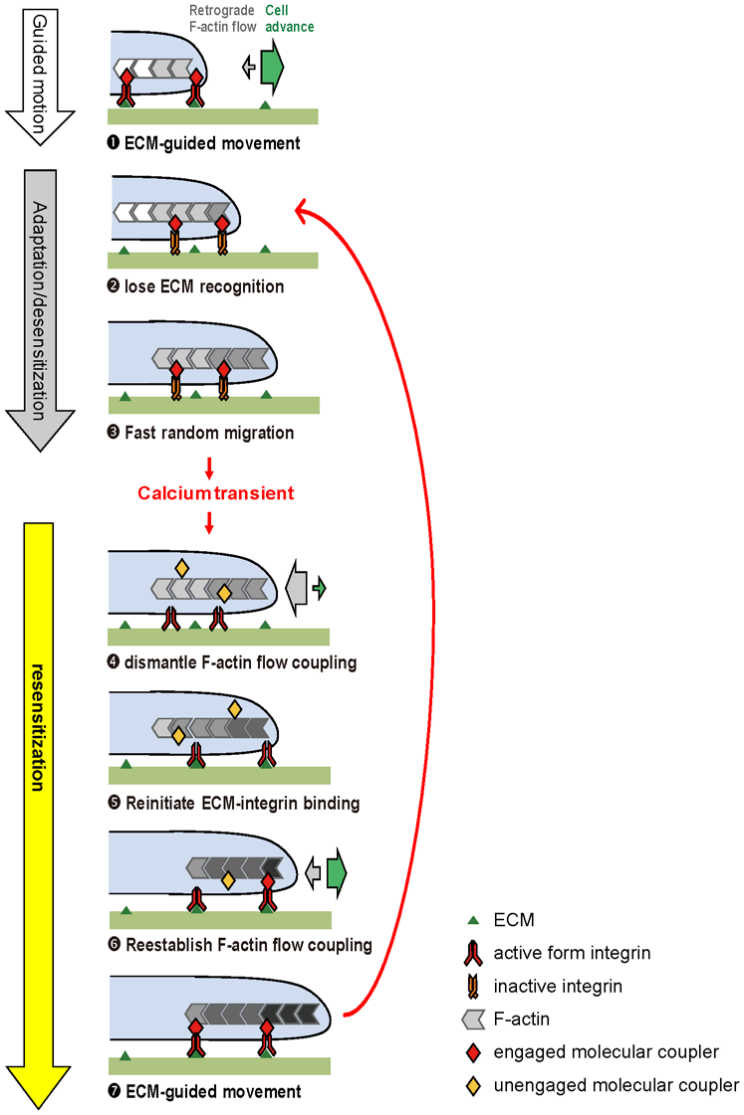
This model describes mechanisms underlying adaptation and calcium-induced resensitization-desensitization cycle for substrate-guided motion. When the cell first encounters a substrate, active-form integrins (*red*) bind to ECM and establish a molecular coupling mechanism (*red diamonds*) that converts retrograde F-actin flow into cell advance. This cell undergoes substrate-guided motion and exhibits minimal retrograde F-actin flow 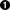. Over time, the integrins become inactive (*orange*) and lose their binding to the substrate 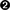. This cell is now adaptive and desensitized to ECM guidance; it still moves fast but in random directions 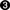. A major calcium transient can dismantle the molecular coupler of F-actin flow (*yellow diamonds*), resulting in a halt of cell motion and appearance of retrograde F-actin flow 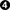. Subsequent calcium signaling then reactivates integrins (*red*) and reinitiates their binding to ECM 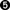. This reestablishes F-actin flow coupling according to where the integrins are interacting with ECM 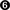, and as a result, the cell is resensitized to substrate guidance and undertakes another period of directed movement 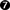. Such navigation soon becomes adaptive again when the cell recommences undirected random migration (back to 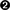). The cycles of resensitization-desensitization repeat as calcium transients oscillate.

The experimental findings presented in this report suggest the model depicted in Fig. 5. When first encountering an ECM substrate in the phase of guided motion, the cell contains active-form integrins and can selectively bind to ECM molecules. Such ECM-integrin binding triggers molecular mechanisms (*red diamond*) that effectively couple retrograde F-actin flow (*gray chevrons*) to the substrate and as a result, fast ECM-guided locomotion occurs 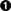. Subsequently, the cell gradually becomes adaptive/desensitized to the substrate guiding ECM, although the fast motion persists. This can occur if the integrins lose their initial high affinity binding to the ECM 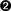 [17] and become inactive (*red to orange heterodimmer*), but the F-actin flow still remains coupled to the ECM substrate (*red diamond*). This cell is now undergoing fast but unguided migration, and exhibiting little retrograde-F-actin flow 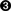. The adaptive/desensitized substrate guiding mechanisms can be reactivated or resensitized by a calcium transient. During such a calcium-induced resensitization process, the calcium transient first dismantles the molecular complex that couples the F-actin flow 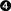 (*red to yellow diamond*), possibly through calcium-dependent protease activity [18]. As a result, the highly coupled F-actin network starts slipping as robust retrograde F-actin flow appears; this is accompanied by a slowing of forward cell movement. The signaling associated with the calcium transient then reactivates the integrin receptors [19] and reinitiates their bindings to ECM ligands 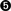, and by doing so, reactivates the recognition of the ECM navigation signals. The downstream integrin signaling also reestablishes the coupling between F-actin and the substrate (*yellow to red diamonds*) 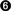 that progressively reduce the slippage of the F-actin flow. As a result, the cell resumes a guided forward movement navigated by the spatial pattern of ECM deposition on the substrate 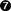. The restoration of ECM-guided movement is only transient, as the guided motion soon becomes adaptive/desensitized. The cycle then repeats.

For most sensing mechanisms, being able to quickly adapt to a steady-state environmental cue is advantageous in that detection can be adjusted to become more sensitive to the changes, rather than absolute amount, of the modulator [20]; this increases the dynamic range of sensing. To make the system work, the adaptive sensing needs to be periodically reactivated so the cell can readjust itself to its ever-changing environment [21]. We propose here that the adaptation of cell movement to ECM-guidance can be achieved by inactivating the integrins after their initial binding/recognition to ECM, while maintaining the motion generation machinery that continues to convert retrograde F-actin flow to forward cell movement. The adaptive motility can periodically be reactivated through reactivation of the integrins and this occurs by spontaneous calcium oscillations, which are typical of motile cells [22]. Previous investigations on the role played by calcium signaling in cell motility have reveal complex and sometime contradictory views [23]. In some cases, increases in intracellular calcium can be anti-migratory and are associated with cell detachment [24], whereas in other cases, calcium signaling increases cell adhesiveness as a prelude to cell motion [25]. We propose here a novel view of calcium regulation of cell motility whereby a major calcium transient may *reset* the cellular response to the outside motogenic cues and allow the cell to restart the sensing of the environment. Whether the resulting response is a promotion or an inhibition of locomotion will depend on how the signals are integrated during the limited sensing time window before the cell becomes adaptive to the guiding cues.

## Materials and Methods

### Cell culture

Fish keratocytes were prepared from the scales of goldfish (*Carasius auratus*) using the technique reported by Lee et al. [26]. Briefly, the scales were removed from the fish and plated on 22×22 mm coverslips drenched with medium (Phenol red-free DMEM supplemented with 10% FCS, 25 mM Hepes, 2 mM L-glutamine and 100 U/ml penicillin/streptomycin); then they were incubated at room temperature to allow keratocytes to migrate out of the scales. After 24 h, the scales were removed and the coverslips were washed with phosphate-buffered saline (PBS) and treated with 0.05% trypsin-EDTA to harvest the cells, which were re-seeded back onto the substrates for the experiments.

### Fabrication of FBN-patterned substrate

The substrate with FBN trails was fabricated using micro-contact printing techniques as previously described [27]. The template wafer contained linear features that were 10 µm wide and 30 or 40 µm apart; these were first created by photolithography in the core facility of National Nano Device Laboratories, Sinchu, Taiwan. Etching was typically 150-nm in depth. PDMS (Poly-dimethyl siloxane, Sil-More industrial Ltd.) was mixed with elastomer and hardener at a 10∶1 ratio, degassed in vacuum and then poured over to the template wafer. After curing at 60°C for 4 h, the PDMS stamp was carefully separated from the template wafer, which was treated with oxygen plasma to render the surface hydrophilic, inked with 50 µg/ml fibronectin (Sigma Aldrich), then printed onto a pre-washed coverslip. The printing quality was examined by adding 1 µg/ml BSA-TRITC (Invitrogen) to the FBN ink solution, which was followed by observation under a fluorescence microscope.

### Calcium uncaging and calcium imaging

Fish keratocytes were first loaded with the caged calcium compound (10 µM NP-EGTA AM, Invitrogen) and a calcium indicator (3 µM Calcium green-1 AM or 5 µM Fura-red AM, for intensity and ratio imaging, respectively) in serum-free medium for 30 min, washed 3 times with culture medium and subjected to uncaging (photo-activation) and recording using a fluorescence microscope equipped with an ORCA-ER CCD camera (Hamamatsu) or by Olympus FV-1000 or Leica SP5 confocal microscopy. Wide-field photoactivation was done using a 10x NA 0.30 objective and a dichroic mirror (11000v3, Chroma) that selected 350 nm UV generated by a 100 W mercury lamp. Focused photoactivation was done by 60x NA 1.25 oil emersion objective mounted on an Olympus FV-1000 confocal microscope illuminated by 405 nm diode-laser. A focused spot of 2 µm in diameter was created for local calcium photo-release. Calcium imaging based on intensity or ratio measurements was performed using methods described previously [28].

### Interference reflection microscopy

Interference reflection microscopy (IRM) was used to examine the dynamic interactions of live cells with the (glass) substrate [29]. IRM was set up using a Leica SP5 confocal microscope or an inverted microscope (DM-IRBE, Leica) equipped with a 50/50 beam splitter and a 63x NA 1.32 oil immersion objective. In the latter case, time-lapsed recording was done by ORCA-ER CCD camera and analyzed by the MetaMorph program (Molecular Devices).

### Immuno-fluorescence staining

Cells were fixed with 4% paraformaldehyde in PBS for 15 min, permeabilized with 0.5% Triton X-100 in fixative for 5 min, blocked with 0.5% BSA in PBS for 30 min, then incubated with primary antibodies in 1% BSA/PBS at 4°C overnight. The antibody probes applied included 5 µg/ml anti-human β1 integrin active conformations monoclonal antibody (Chemicon), 2 µg/ml anti-phosphotyrosine clone 4G10 (Upstate) and 20 µg/ml anti-phospho-paxillin tyr118 antibody (Cell signaling). After PBS washes, secondary antibodies (30 µg/ml goat anti-mouse or -rabbit IgG conjugated with FITC, Jackson ImmunoResearch) were applied at room temperature for 1 h. Cells were mounted in anti-photobleaching medium and observed by fluorescence or confocal microscopy.

### Bead preparation

The polystyrene beads with a diameter of 600 nm (Bangs Laboratories, Inc.) were coated with polyethyleneimine (Sigma) and incubated overnight at 4°C. The beads were replaced in culture medium supplemented with 10 mg/ml bovine serum albumin before application to the cells.

### Western blotting

Cells were lysed and extracted with RIPA solution (20 mM Tris-HCl pH 7.5, 150 mM NaCl, 1 mM EDTA, 1 mM EGTA, 1% NP-40, 1% sodium deoxycholate and 1 mM Na_3_VO_4_) supplement with protease inhibitor cocktails (Merck). Proteins were separated by SDS-PAGE and transferred to polyvinylidene difluoride (PVDF) membrane (Millipore). After blocking with 5% BSA in Tris-buffered saline with 0.1% Tween 20 (TBST) at room temperature for 1 h, the membrane was incubated with the primary antibody, which included 1 µg/ml anti-phosphotyrosine clone 4G10, 1 µg/ml anti-phospho-paxillin tyr118 antibody, 0.5 µg/ml anti-paxillin antibody (BD Transduction Laboratories) and 0.5 µg/ml anti-actin AC-40 antibody (Sigma), at room temperature for 2 h. The membrane was then washed three times with TBST and incubated with horseradish peroxidase-conjugated secondary antibody (Thermo Scientific). The blotting signals were visualized using SuperSignal West Femto Maximum Sensitivity Substrate (Thermo Scientific).

## Supporting Information

Movie S1 Fish keratocytes are noted to leave the FBN trail after initial adherence and movement along the FBN paths. A calcium transient created by uncaging at 0:00 is noted to reinitiate a period of ECM-guided motion. Photographs are taken every 30 sec. Time (min:sec) after cell plating is shown. Bar = 100 µm.
(3.27 MB MOV)

Movie S2 DIC and calcium imaging sequences reveal reorientation of cell movement on a homogenous FBN substrate after the cell receives calcium uncaging at time zero. Photographs are taken every 3 sec. Bar = 10 µm.
(2.27 MB MOV)

Movie S3 Mock photolysis did not influence either the rate or direction of the ongoing cell movement. Time (min:sec) after mock photolysis is shown. Photographs are taken every 3 sec. Bar = 10 µm.
(3.26 MB MOV)

Movie S4 The IRM image sequence of a cell receiving calcium uncaging treatment. The cell immediately detaches from the substrate after the illumination as evidenced by the increased IRM brightness, followed by rebinding and crawling of the cell along the FBN path accompanied by IRM darkness at the leading cell lamella. Photographs are taken every 5 sec. Bar = 10 µm.
(1.91 MB MOV)

Movie S5 The IRM image sequence of a cell exposed to MnCl_2_. A progressively increased cell adhesion is evidenced by the increase of IRM darkness. Such abnormally tight cell-substrate association is inhibitory for cell motility. Photographs are taken every 5 sec. Bar = 10 µm.
(2.70 MB MOV)

Movie S6 The IRM image sequence of a cell exposed to DTT. Similar increases of cell-substrate association and IRM darkness, as that caused by MnCl_2_, are noted. And as a result, cell motility is progressively inhibited. Photographs are taken every 5 sec. Bar = 10 µm.
(2.97 MB MOV)
